# Neuroimaging in PRES: Isolated Brainstem and Spinal Cord Involvement Secondary to Aorto-Iliac Occlusive Disease—A Unique Case Report

**DOI:** 10.5334/jbsr.4015

**Published:** 2025-09-12

**Authors:** Lucas Dekesel, Laura Wuyts, Pieter Olivier

**Affiliations:** 1 AZ Sint-Lucas, Ghent, Belgium

**Keywords:** posterior reversible encephalopathy syndrome (PRES), hypertensive encephalopathy, PRES with spinal cord involvement (PRES-SCI), brainstem PRES, aorto-iliac occlusive disease

## Abstract

*Teaching point:* Consider atypical PRES and a vascular work-up in brainstem edema, especially in the setting of severe hypertension.

## Introduction

Posterior reversible encephalopathy syndrome (PRES) is a clinico-radiological condition characterized by acute neurological symptoms and vasogenic edema on magnetic resonance imaging (MRI). While typically affecting the parieto-occipital regions, PRES may also involve the cerebellum and brainstem. Rarely, PRES extends into the spinal cord, referred to as PRES with spinal cord involvement (PRES-SCI) [1]. This case report presents an unusual case of PRES-SCI confined to the brainstem and cervicomedullary junction, secondary to aorto-iliac occlusive disease.

## Case Presentation

**Figure 1:**
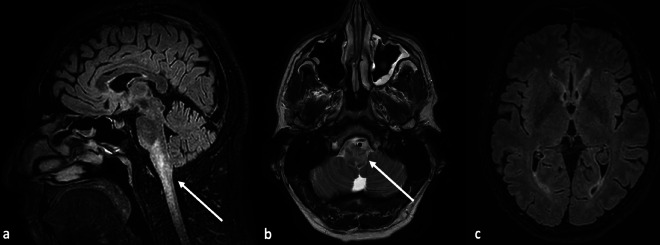
Sagittal FLAIR **(a)** and axial T2-weighted images **(b)** showing edematous changes in a swollen brainstem at the level of the pons and medulla oblongata, extending further in the cervical spinal cord (arrows). There was no pathological diffusion restriction or contrast-enhancement (not shown), in keeping with vasogenic edema. Note the absence of edematous changes supratentorially, with a normal appearance of the basal ganglia and subcortical white matter **(c)**.

A 44-year-old man presented with a one-week history of worsening headache and visual disturbances. Neurological examination showed no focal signs. Blood pressure was markedly elevated, with systolic blood pressure of more than 200 mmHg. Ophthalmologic examination revealed severe hypertensive retinopathy with cotton wool spots. Brain MRI revealed vasogenic edema in the lower brainstem and cervicomedullary junction (Figure 1). Notably, no supratentorial involvement, diffusion restriction, or contrast enhancement was observed. An atypical form of PRES was suspected, though malignancy and inflammatory conditions such as myelitis or neuromyelitis optica spectrum disorder (NMOSD) were also considered.

**Figure 2:**
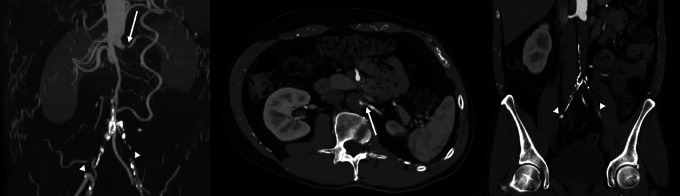
CT angiogram with virtual reconstruction showing aorto-iliac occlusion with ostial stenosis of the left renal artery (arrows) and re-injection distal into the common iliac arteries (arrowheads).

The patient’s hypertension was refractory to initial therapy. CT angiography of the renal arteries revealed severe ostial stenosis of the left renal artery secondary to aorto-iliac occlusion (Leriche syndrome) (Figure 2). The patient underwent renal artery stenting, leading to blood pressure normalization and symptom resolution.

**Figure 3:**
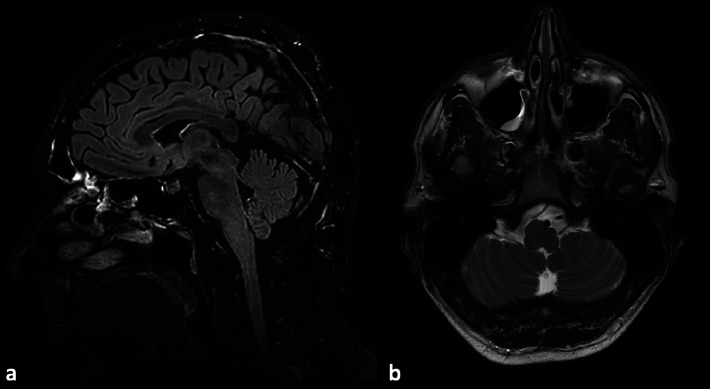
Sagittal FLAIR **(a)** and axial T2-weighted follow-up images **(b)** showing complete resolution of the vasogenic edema in the brainstem and cervical spinal cord without atrophy or residual changes.

Follow-up MRI at eight weeks showed complete resolution of edema without residual lesions or atrophy (Figure 3). Serology for AQP-4 and MOGAD antibodies was negative. Given the full resolution on MRI following blood pressure control and the absence of evidence for other pathologies, the diagnosis of PRES-SCI was established.

## Discussion

PRES is believed to result from impaired cerebrovascular autoregulation leading to vasogenic edema in the context of severe hypertension, typically posterior in the cerebral hemispheres. However, PRES-SCI is a rare presentation, with brainstem and spinal cord involvement likely due to dysregulation in the vertebrobasilar circulation [1]. To our knowledge, this is the first reported case associating PRES-SCI with aorto-iliac occlusive disease. The rapid clinical and radiological recovery following blood pressure normalization underscores the importance of vascular evaluation in atypical PRES presentations. Considering PRES-SCI in the differential diagnosis of brainstem and spinal cord edema could prompt appropriate management.
